# Genome-wide association and epistatic interactions of flowering time in soybean cultivar

**DOI:** 10.1371/journal.pone.0228114

**Published:** 2020-01-22

**Authors:** Kyoung Hyoun Kim, Jae-Yoon Kim, Won-Jun Lim, Seongmun Jeong, Ho-Yeon Lee, Youngbum Cho, Jung-Kyung Moon, Namshin Kim

**Affiliations:** 1 Genome Editing Research Center, Korea Research Institute of Bioscience and Biotechnology (KRIBB), Daejeon, Republic of Korea; 2 Department of Bioinformatics, KRIBB School of Bioscience, University of Science and Technology (UST), Daejeon, Republic of Korea; 3 National Institute of Agricultural Sciences, Rural Development Administration, Jeonju, Republic of Korea; University of Guelph, CANADA

## Abstract

Genome-wide association studies (GWAS) have enabled the discovery of candidate markers that play significant roles in various complex traits in plants. Recently, with increased interest in the search for candidate markers, studies on epistatic interactions between single nucleotide polymorphism (SNP) markers have also increased, thus enabling the identification of more candidate markers along with GWAS on single-variant-additive-effect. Here, we focused on the identification of candidate markers associated with flowering time in soybean (*Glycine max*). A large population of 2,662 cultivated soybean accessions was genotyped using the 180k Axiom^®^ SoyaSNP array, and the genomic architecture of these accessions was investigated to confirm the population structure. Then, GWAS was conducted to evaluate the association between SNP markers and flowering time. A total of 93 significant SNP markers were detected within 59 significant genes, including *E1* and *E3*, which are the main determinants of flowering time. Based on the GWAS results, multilocus epistatic interactions were examined between the significant and non-significant SNP markers. Two significant and 16 non-significant SNP markers were discovered as candidate markers affecting flowering time via interactions with each other. These 18 candidate SNP markers mapped to 18 candidate genes including *E1* and *E3*, and the 18 candidate genes were involved in six major flowering pathways. Although further biological validation is needed, our results provide additional information on the existing flowering time markers and present another option to marker-assisted breeding programs for regulating flowering time of soybean.

## Introduction

A genome-wide association study (GWAS) is one of the promising approaches for the identification of genomic variants responsible for specific phenotypes [1]. With the introduction of high-density marker arrays, GWAS has been actively used in many crop species [2] and has enabled the discovery of single nucleotide polymorphism (SNP) markers associated with numerous agronomic traits [3]: flowering time [4, 5], cold tolerance [6], salt tolerance [7, 8], drought tolerance [9], disease resistance [10, 11], plant height [12, 13], leaf architecture [14, 15], and seed weight [16, 17]. The knowledge of the trait-related SNP markers has served as the genetic basis for the improvement of various traits in crop breeding programs [18].

Days to flowering (DTF) is a crucial agronomic trait that regulates the maximum use of sunlight and temperature [19], and affects the growth and yield potentials [20]. Many GWAS on single-variant-additive-effect thus have carried out to improve yield productivity, and have led to identify DTF markers in many crops. For example, three candidate genes including *Nsn1*, *Fpa*, and *Zmm22* were identified in 942 maize samples (*Zea mays*) [21]; two candidate genes, *CO1* and *BFL*, were identified in using 218 barley samples (*Hordeum vulgare*) [22]; eight candidate genes including *Hd1* were confirmed in 950 rice samples (*Oryza sativa*) [23]; and ten candidate genes including *SOC1*, *AGL6*, and *ELF8* were reported in 309 soybean samples (*Glycine max*) [13]. These findings have presented valuable information to various breeding programs focused on DTF, but have a limitation to further improve DTF, because they are not sufficient for explaining all of the phenotypic variations in DTF such as interaction effects between markers [24].

Epistasis is defined as the interaction between genes or SNP markers that influences a trait [25]. Each SNP marker above a significant level in GWAS has a strong effect on the determination of a trait, but non-significant markers that interact with each other could also have a large influence on the trait [24]. Therefore, considering epistatic interactions for multi-variant non-additive effects, enables to discover more markers associated with traits, together with GWAS on single-variant-additive-effects [26]. For this reason, many GWAS studies have utilized epistatic analysis as a complementary approach, and have reported significant epistasis and GWAS markers associated with various traits: 12 epistatic markers with 12 GWAS markers for sudden death syndrome resistance in soybean [27], ten epistatic markers with 33 GWAS markers for iron deficiency-related chlorosis in soybean [28], nine epistatic markers with 14 GWAS markers for seed weight in soybean [29], and 38 epistatic markers with 113 GWAS markers for DTF in barley [30]. Significant markers controlling many traits have been investigated through these studies; however, soybean’s DTF markers related to both epistasis and GWAS have not been investigated in depth as other traits.

Twelve major genes affecting DTF have been identified in soybean, including *E1–E10* [31–41], *J* [42], and *Dt1* [43, 44]. To better understand the DTF-related genetic factors beyond the twelve major genes, an epistasis study with GWAS is needed. Here, we genotyped 2,662 cultivated soybean accessions using the high-density Axiom^®^ 180k SoyaSNP array developed by our team in 2016 [45]. Also, we evaluated the DTF trait of all accessions through a phenotypic survey. The aims of this study were: 1) to examine population structures of the 2,662 accessions for GWAS; 2) to identify significant markers associated with DTF through GWAS; 3) to reveal epistatic markers with interactions between significant and non-significant GWAS markers; and 4) to present final candidate markers with the relation to major DTF pathways.

## Materials and methods

### Plant materials and genotyping

**Fig 1:**
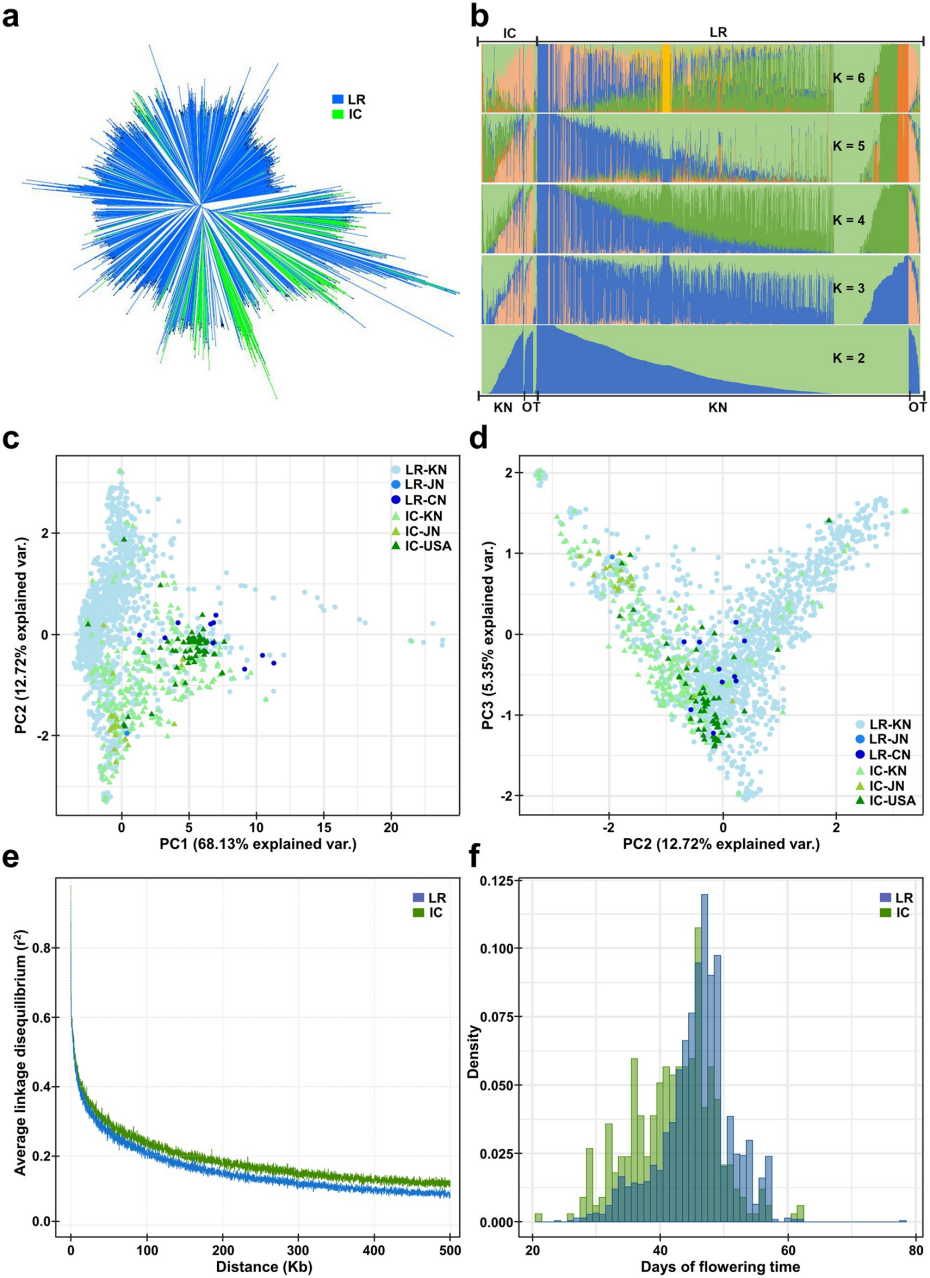
Genomic structure of 2,662 cultivated soybean accessions and their relationship among each other. a. Phylogenetic tree computed using the identical-by-state coefficient. b. Population structure analysis using the number of genetic clusters (*K*) ranging from *K* = 2 to *K* = 6, based on the maximum likelihood-based clustering algorithm. c. Principal component analysis (PCA) plot of PC1 and PC2 derived using the Kimura two-parameter model. d. PCA plot of PC2 and PC3. e. Extent of linkage disequilibrium (LD) decay computed up to 500 kb. f. Distribution of the flowering time of soybean LRs and ICs. Abbreviations, KN, JN, CN, and OT, indicate accessions collected from Korea, Japan, China, and the other regions, respectively.

A total of 2,872 soybean (*Glycine max*) accessions were collected from the National Agrobiodiversity Center in the Rural Development Administration (RDA, Jeonju, Korea), and were genotyped using the Axiom^®^ 180k SoyaSNP. Of these, 210 hybrid accessions were excluded from the analysis. The remaining 2,662 accessions originated from South Korea (2,415), North Korea (96), the USA (60), China (59), and Japan (32), and comprised 335 improved cultivars (ICs), 2,175 landraces (LRs), and 152 unknown cultivars (UCs) (S1 Dataset). These accessions belonged to the following seven maturity groups: I (2), II (31), III (60), IV (187), V (88), VI (16), and VII (3); however, the maturity group of most of the Korean accessions (2,275) was not reported. After genotyping, 180,961 SNP markers were detected, and haplotype phasing and imputation were conducted using BEAGLE version 3.3.1 [46]. Then, 78,427 SNP markers with a minor allele frequency (MAF) > 0.05 were obtained. The non-MAF filtered SNPs were used to examine the genomic structure and relationship, and the MAF filtered SNPs were used to perform the GWAS. The genotype data of all 2,662 accessions, which were generated in collaboration with the RDA, is available at http://k-crop.kr and https://github.com/kyounghyoun/Soybean_epistasis [47]. Phenotypic evaluation was conducted in the experimental field of the National Institute of Crop Science (NICS, Jeonju, Korea) (35°50'26.7" N, 127°02'42.7" E), and the DTF trait was measured from June to October in 2014. Because of an agreement on limited disclosure with our phenotypic evaluation team, the raw DTF values are provided in the distribution plot and categorical data (Fig 1f, S1 Dataset).

### Analysis of the genomic structure of and relationship among soybean accessions

Phylogenetic analysis was conducted based on the identity-by-state coefficient matrix calculated using Plink v1.90b [48]. The phylogenetic tree was reconstructed using the BIO-neighbor-joining algorithm [49] and FigTree v1.4.3 (http://tree.bio.ed.ac.uk/software/figtree/) (Fig 1a). Structure analysis was conducted using fastSTRUCTURE v1.0 [50], based on a variational Bayesian framework (Fig 1b). The number of genetic clusters (*K*) was set at six, considering the error values of 10-fold cross-validation (S1 Fig). The error value was the lowest at *K* = 6. Principal component analysis (PCA) was performed by applying singular value decomposition to the distant matrix calculated using the Kimura two-parameter model [51], and then displayed using the PC axes 1, 2, and 3 (Fig 1c and 1d). The linkage disequilibrium (LD) pattern was computed using PopLDdecay v3.2 [52]. The mean value of LD was calculated within 100 and 500 kb regions (S1 Table), and the degree of LD up to 500 kb is displayed in Fig 1e. Nucleotide diversity (π) was calculated using a 10 kb slide size with a 100 kb window size using VCFtools v4.2 [53], and inbreeding coefficient (F) was estimated using the method of moments in VCFtools (S1 Table). Additionally, using the same software, fixation index value (Fst) was calculated using a 10 kb slide size with a 100 kb window size.

### GWAS

**Fig 2:**
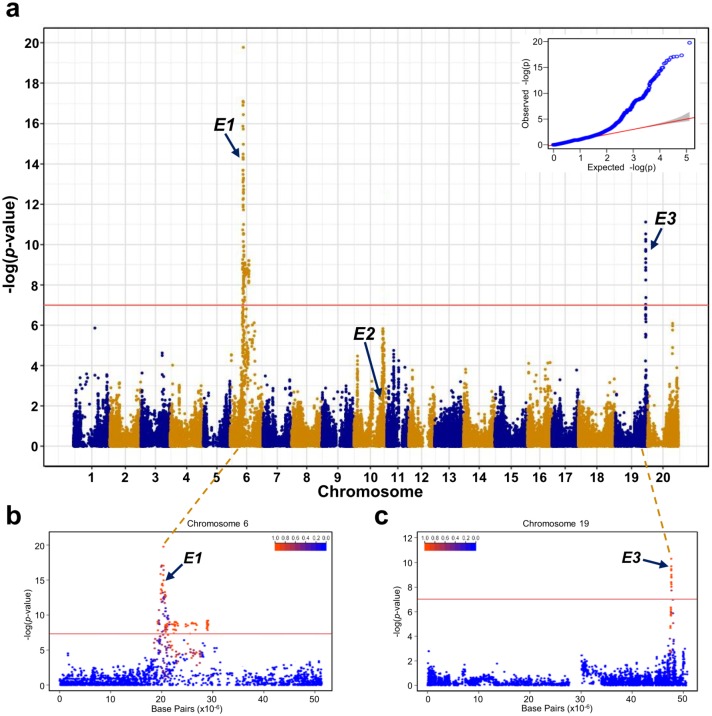
Manhattan plot for the DTF trait of soybean accessions. a. Manhattan plot of all 20 chromosomes. b. Manhattan plot of chromosome 6 harboring the *E1* gene. c. Manhattan plot of chromosome 19 harboring the *E3* gene. Red horizontal lines indicate the statistically significant cut-off of −log(*p*-value) = 7.

An association study between genomic regions and flowering time was conducted using a compressed mixed linear model within Genomic Association and Prediction Integrated Tool [54]. The MAF-filtered 78,427 SNP markers (MAF > 0.05) was used to consider common markers (S2 Dataset), and a statistically significant cut-off value was adopted to −log(*p*-value) = 7. To minimize false-positive results, the cut-off was set slightly higher than the Bonferonni-corrected −log(*p*-value) of 6.2. The extent of model fitting was confirmed using a quantile–quantile (Q-Q) plot for the expected and obtained *p*-values. After GWAS based on SNP markers, gene annotations were performed using SnpEff v4.3 [55]. All results are summarized in S3 Dataset, and genome-wide plots including the Q-Q plot are shown in Fig 2.

### SNP marker set for epistatic analysis

To construct an SNP marker-set for multilocus epistatic interaction analysis, a biological filtering approach was conducted. First, genes affecting DTF and maturity in *Arabidopsis* were examined [56–59], and a total of 356 *Arabidopsis* genes were identified (Table 1, S4 Dataset). Second, nucleotide sequences of these *Arabidopsis* genes were mapped onto the soybean reference genome Wm82.a2.v1 using tBLASTx [60] with matching identity >80% and alignment length >70 bp options, and a total of 2,643 homologous soybean genes were identified. Third, 2,188 genes belonging to the Axiom^®^ 180k SoyaSNP array were identified among the 2,643 soybean genes, and 5,447 SNP markers contained in the 2,188 genes were obtained. Finally, the 5,447 SNP markers were combined with 93 significant genic SNP markers derived in the GWAS (-log(*p*-value) > 7), and a total of 5,534 SNP markers (excluding 6 overlapping SNP markers) was constructed as the final SNP marker-set for the multilocus epistatic analysis. All results, including the number of SNP markers per gene, are summarized in S4 Dataset, and a brief summary is provided in Table 1.

**Table 1:** Significant genes related to DTF derived from GWAS, and non-significant genes related to DTF and maturity in *Arabidopsis*.

DTF	Number of Arabidopsis genes	Corresponding soybean genes	Genes on the 180k SoyaSNP array	SNP markers on the 180k SoyaSNP array
GWAS^a^	55	59	59	93
Related genes^b^	353 (3)	2,639 (4)	2,184 (4)	5,441 (6)
Total	408	2,698	2,243	5,534

^a^Significant genes related to DTF derived from GWAS

^b^Non-significant genes related to DTF and maturity in *Arabidopsis*. Values in parenthesis indicate the number of genes that overlapped with significant genes in GWAS.

### Multilocus epistatic interactions

**Fig 3:**
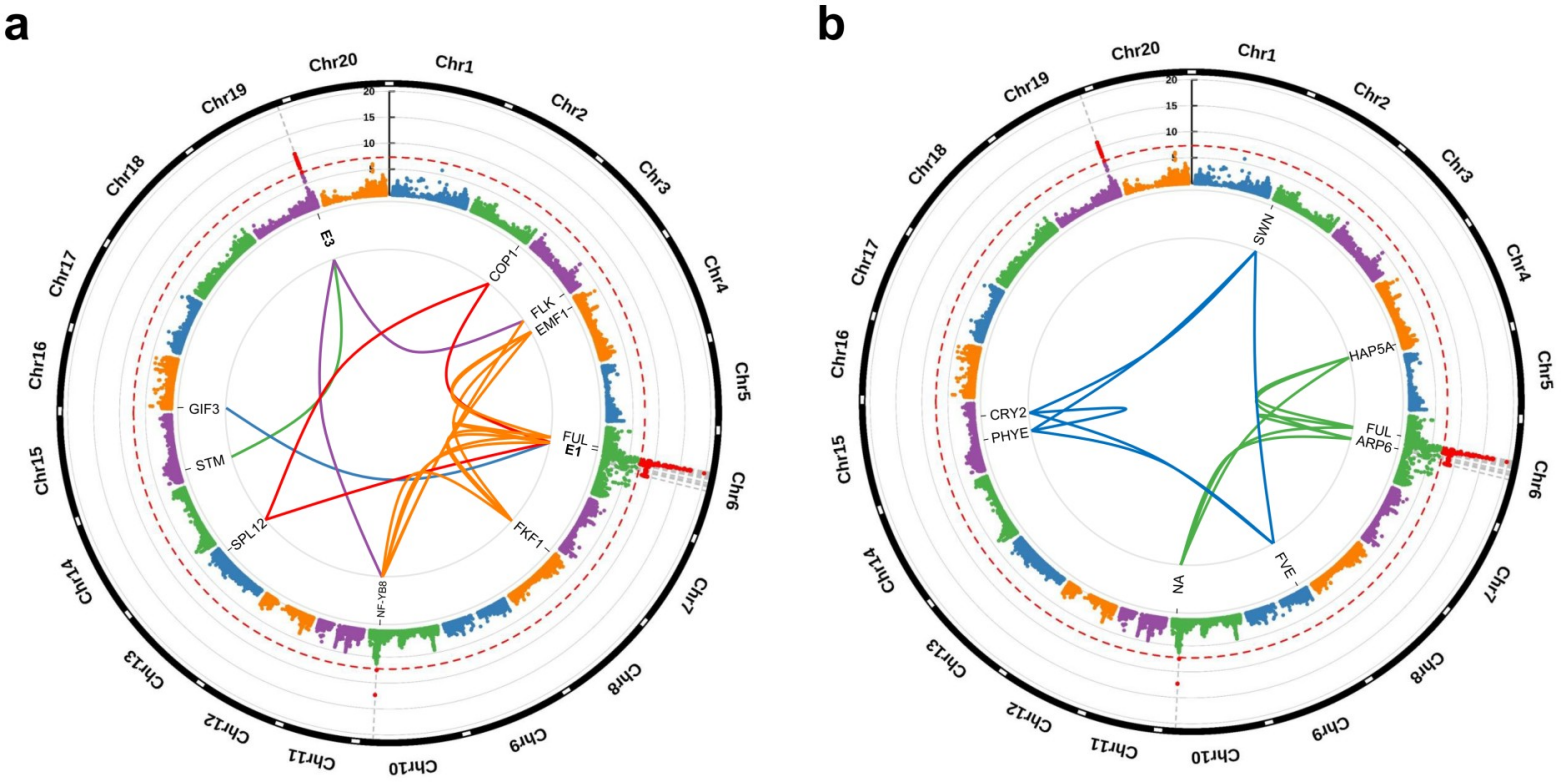
Epistatic interactions among DTF-related genes. a. Epistatic interactions between non-significant genes and significant genes (*E1* and *E3*). Inner curved lines indicate interaction groups, G1 (navy), G2 (red), G3 (orange), G4 (green), and G5 (purple) (Table 2). b. Epistatic interaction between non-significant genes. Inner curved lines indicate interaction groups, G6 (green) and G7 (navy) (Table 2). Red dotted lines in the two plots indicate statistically significant cut-off thresholds of −log(*p*-value) = 7.

Multilocus interactions among the 5,534 SNP markers related to DTF were examined using Bayesian High-order Interaction Toolkit (BHIT) [61] with 1,000,000 iterations and 990,000 burn-in periods. The relationship between SNPs (X) and trait (Y) in multilocus interactions was inferred as *P*(*Y*, *H*|*I*, *H*) by grouping dependent genotypes (I) and phenotypes (H) [61]. Then, the likelihood was estimated using the following equation:

$$P(Y,H|I,H)=P(Y|X_{\left\{I=1\right\}})\prod_{h=1}^{H}P(X_{\left\{I=h\right\}}|I)=(\prod_{m=1}^{M}\left(P(Y_{\left\{m\right\}}|X_{\left\{I=1\right\}}=m\right))(\prod_{m=1}^{H}P(X_{\left\{I=h\right\}}|I))$$

where, *X*_{*I*=*h*}_ indicates all X in the *h*^*th*^ group, and Y{m} indicates Y in the *m*^*th*^ cluster. For *P*(*X*_{*I*=*h*}_|*I*), Dirichlet prior distribution and multinomial distribution were used as the Bayesian partition model [62]. The threshold for the posterior probabilities on the dependency for multilocus and phenotype was set at 0.5. A Markov Chain Monte Carlo approach was then used to search potential epistatic marker sets, and a Bayesian computational approach was utilized to detect final high-order epistatic market sets. All results of multilocus epistatic interactions are summarized in Table 2, and displayed in Circular-Manhattan plots in Fig 3. To more complement the multilocus epistasis, two-locus epistatic interactions were investigated to the BHIT results. The two-locus epistatic test was conducted using a fixed linear regression model of the Plink v1.90b [48], and the resulting *p*-values are summarized in Table 2.

**Table 2:** Multilocus epistatic interactions among 5,534 SNP markers related to DTF.

Group^a^	Chr.	Position (bp)	Soybean gene ID	Gene symbol^b^	*p*-value (min, max)^c^	Protein	Quantitative trait loci (QTLs)	Related pathway^d^
A. Significant interaction groups including significant DTF-related genes in GWAS								
G1	6	20,208,679	Glyma.06G207800	*E1	(3.69E-03, 3.69E-03)	AP2/B3-like transcriptional factor family protein	First flower 4–1, 5–1, 12–2, 23–1, 26–9, 26–12	P
	16	1,855,885	Glyma.16G020400	*GIF3*	(3.69E-03, 3.69E-03)	GRF1-interacting factor 3	Pod maturity 9–1	FPI
G2	6	20,207,322	Glyma.06G207800	*E1	(5.36E-03, 8.21E-03)	AP2/B3-like transcriptional factor family protein	First flower 4–1, 5–1, 12–2, 23–1, 26–9	P
	2	45,220,188	Glyma.02G267800	*COP1*	(8.21E-03, 1.56E-02)	Transducin family protein/WD-40 repeat family protein	-	P
	13	37,658,295	Glyma.13G274900	*SPL12*	(5.36E-03, 1.56E-02)	Squamosa promoter-binding protein-like 12	Plant height 38–2	FMI
G3	6	20,207,322	Glyma.06G207800	*E1	(3.97E-04, 7.40E-01)	AP2/B3-like transcriptional factor family protein	First flower 4–1, 5–1, 12–2, 23–1, 26–9, 26–12	P
	4	6,893,070	Glyma.04G081800	*EMF1*	(1.27E-25, 7.25E-02)	Embryonic flower 1	-	V
	6	19,585,253	Glyma.06G205800	*FUL*	(2.61E-04, 9.17E-02)	AGAMOUS-like 8	First flower 4–1, 5–1, 12–2, 26–9, 26–12	FMI
	8	3,655,383	Glyma.08G046500	*FKF1*	(1.27E-25, 2.26E-02)	Flavin-binding, kelch repeat, f box 1	-	P
	10	42,450,765	Glyma.10G192000	*NF-YB8*	(3.97E-04, 9.17E-02)	Nuclear factor Y, subunit B8	Pod maturity 14–2, 15–2	FPI
G4	19	47,638,344	Glyma.19G224200	*E3	(1.84E-03, 1.84E-03)	Phytochrome A	First flower 4-g80	P
	15	8,774,152	Glyma.15G111900	*STM*	(1.84E-03, 1.84E-03)	KNOX/ELK homeobox transcription factor	First flower 12–3	P
G5	19	47,638,344	Glyma.19G224200	*E3	(2.46E-06, 5.94E-03)	Phytochrome A	First flower 4-g80	P
	3	44,468,848	Glyma.03G248200	*FLK*	(4.85E-03, 5.94E-03)	RNA-binding KH domain-containing protein	Plant height 26–17	AU
	10	42,457,595	Glyma.10G192000	*NF-YB8*	(2.46E-06, 4.85E-03)	Nuclear factor Y, subunit B8	Pod maturity 14–2, 15–2	FPI
B. Significant interaction groups among non-significant DTF-related genes								
G6	4	46,807,590	Glyma.04G196200	*HAP5A*	(3.89E-10, 1.82E-01)	Nuclear factor Y, subunit C1	Plant height 5–4; Pod maturity 6–3, 6–6, 32–1	FPI
	6	26,630,946	Glyma.06G221000	*ARP6*	(1.64E-03, 2.52E-01)	Actin-related protein 6	First flower 3–1, 8–1, 22–2, 23–1, 26–10, 26–11, 26–13, 26–16	AU
	6	19,586,329	Glyma.06G205800	*FUL*	(3.89E-10, 2.53E-06)	AGAMOUS-like 8	First flower 4–1, 5–1, 12–2, 26–9, 26–12	FMI
	10	49,716,656	Glyma.10G274300	*BBX15*	(2.53E-06, 2.52E-01)	B-box type zinc finger protein with CCT domain	-	P
G7	2	1,109,049	Glyma.02G012100	*SWN*	(1.04E-03, 2.20E-01)	SET domain-containing protein	-	AU, V
	9	6,040,554	Glyma.09G063100	*FVE*	(2.13E-12, 1.04E-03)	Transducin family protein/WD-40 repeat family protein	-	AM, AU
	15	22,687,645	Glyma.15G196500	*PHYE*	(1.93E-04, 4.36E-01)	Phytochrome E	Pod maturity 31–2, 37–2	P
	15	40,764,606	Glyma.15G223700	*CRY2*	(2.13E-12, 4.36E-01)	Cryptochrome 2	Plant height 26–10	P

*Major DTF-related genes (*E1* and *E3*) identified from GWAS.

^a^Interaction group derived from multilocus epistatic analysis for DTF.

^b^Representative gene symbol corresponding the soybean gene.

^c^Range of minimum and maximum *p*-values for two-locus interactions, calculated pairwisely among SNP markers in an interaction group.

^d^DTF-related pathways: AM, ambient temperature; AU, autonomous; FPI, floral pathway integrator; P, photoperiod; FMI, floral meristem identity; V, vernalization

## Results

### Genomic structure of cultivated soybeans

A large data set of 2,662 cultivated soybean accessions, originated from Korea (2,511), the USA (60), China (59), and Japan (32), were used in this study. These accessions included 335 ICs, 2,175 LRs, and 152 UCs (S1 Dataset). Korean accessions accounted for approximately 94.33% of the entire soybean collection, and covered with most of the soybean accessions in Korea. These 2,662 accessions were genotyped using the Axiom^®^ 180k SoyaSNP array developed by our team in 2016 [45], and a total of 180,961 SNP markers were identified. These SNP markers represented 39,674 soybean genes, with an average number of 2.80 SNP markers per gene. Prior to GWAS, the genomic structure of 2,510 accessions (2,175 LRs and 335 ICs, excluding 152 UCs) was examined using the SNP markers (Fig 1). These 2,510 accessions represented a single large population with considerable genomic diversity. A phylogenetic tree was constructed to radial forms rather than branched forms with distinct clades, and LRs and ICs exhibited a mixed aspect with each other (Fig 1a). Structure analysis revealed similar genomic composition and proportion between LRs and ICs at *K* = 2 (Fig 1b; green and blue). With the increase in the value of *K*, the entire IC group showed a unique genomic composition, which slightly distinguished from the LR group; however, each accession in the IC group exhibited an aspect of inclusion in the LR group, with varying levels of genomic composition. The results of PCA showed that the LR and IC groups formed a single group with large diversity, and the IC group overlapped with the LR group, consistent with the results of structure analysis (Fig 1c and 1d). Average values of LD, π, and DTF showed differences between the IC and LR groups. Due to the impact of conscious selection for improvement, the IC group showed slightly higher average values of LD (0.121583) (Fig 1e, S1 Table), and lower π (2.22E-5) and DTF (42.16) (Fig 1f, S1 Dataset) than the LR group (0.112253, 2.39E-5, and 45.86, respectively). Values of inbreeding coefficient were all high (IC, F = 0.9710; LR, F = 0.9731) because of the characteristics of inbreeding and stringent cleistogamy (S1 Table). Also, a numeric value Fst, supported the results of various population analyses. The Fst between the IC and LR groups was 0.0581 and was small enough to be regarded as a single group. Based on these results, a single dataset consisting of all 2,662 accessions was constructed, and used for subsequent GWAS and epistatic analysis. The results of PCA indicated that 132 UCs overlapped between the LR and IC groups; these accessions were included in GWAS to reflect as much genomic diversity of cultivated accessions as possible.

### GWAS of DTF

Of the 180,961 SNP markers identified in 2,662 accessions, 78,427 SNP markers with MAF > 0.05 were selected as common SNP markers for GWAS. These SNP markers were distributed on all 20 soybean chromosomes and represented 26,162 genes, with an average number of 2.07 SNP markers per gene (S2 Dataset). Then, the association between these SNP markers and DTF was examined using a compressed mixed linear model that adjusts population structure and kinship (Fig 2, S3 Dataset). Statistically significant cut-off was set at −log(*p*-value) = 7, which was higher than the Bonferroni-adjusted *p*-value = 6.2, to minimize false-positive results. A total of 132 significant SNP markers including 93 genic SNP markers were detected, and all mapped to chromosomes 6 and 19 (Fig 2a). Among these 93 significant genic SNP markers, 78 SNP markers mapped to 51 soybean genes on chromosome 6 (Fig 2b), and 15 SNP markers mapped to eight genes on chromosome 19 (Fig 2c).

The two major DTF-related genes, *E1* and *E3*, were identified from the 59 significant genes. The *E1* gene has the largest influence on the determination of DTF [37, 63] and affects vegetative development by regulating multiple genes related to plant growth [64]. The *E1* gene was located at the 20,207,322 bp position on chromosome 6 (Glyma.06G207800), and showed a considerably significant −log(*p*-value) of 14.23. The *E3* gene regulates DTF under short-day and long-day conditions, and affects plant maturity [65]. This gene was positioned at 47,638,344 bp on chromosome 19 (Glyma.19G224200) and exhibited a significant −log(*p*-value) of 9.75. Two additional major genes affecting DTF, *E2* and *E4* [66, 67], were not detected as significant genes in this study. The *E2* gene was located at nucleotide positions 45,295,453, 45,295,508, 45,296,750, and 45,300,271 bp on chromosome 10 (Glyma.10G221500), but all showed non-significant *p*-values of 2.00, 2.43, 2.31, and 2.43, respectively (Fig 2a). Also the *E4* gene was positioned at 33,236,286, 33,236,286, and 33,241,589 bp on chromosome 20 (Glyma.20G090000), but all were excluded from the GWAS because of low MAF (0.000187, 0.000909, and 0.002441, respectively).

### Epistatic interaction among DTF-related genes

Interaction effects on DTF among significant SNP and non-significant SNP markers were examined to complement GWAS single-variant-additive-effect. Significant SNP markers consisted of 93 genic SNP markers belonging to 59 soybean genes derived from the GWAS, as described above. To focus on interactions among DTF-related genes, 5,441 non-significant SNP markers in 2,184 soybean genes (Table 1) were selected from a set of 356 DTF-related *Arabidopsis* genes on the Axiom^®^ 180k SoyaSNP array (S4 Dataset) (see Materials and methods for details). In the 356 *Arabidopsis* genes, a total of 2,188 homologous soybean genes were identified, but four genes (Glyma.06G205700, Glyma.06G207800-*E1*, Glyma.06G221000, and Glyma.19G224200-*E3*) with six SNP markers were excluded from the non-significant SNP marker-set since these markers were already included in the significant marker-set in GWAS. Also, when detecting homologous genes, the non-MAF filtered SNPs were used in order to consider various DTF-related genes which were excluded from the GWAS due to MAF < 0.05, such as *E4* gene (Glyma.20G090000). Finally, a total of 5,534 SNP markers belonging to 2,243 soybean genes were selected and used for analyzing multilocus epistatic interactions. The results revealed five interaction groups (G1–G5) containing two significant and nine non-significant genes (Fig 3a) and two interaction groups (G6, G7) containing only eight non-significant genes (Fig 3b, Table 2).

The interaction groups G1, G2, and G3 contained two, three, and five genes, respectively, each including the significant DTF-related *E1* gene (Fig 3a, Table 2). The non-significant genes, identified in GWAS, included *GIF3* (belonging to G1 group and quantitative trait locus (QTL) of pod maturity 9–1), which is involved in regulating cell expansion and meristem of leaves [68]; *SPL12* (G2, plant height QTL 38–2), which affects plant growth and development [69]; *COP1* (G2), which regulates photomorphogenesis and skotomorphogenes, and is related to growth and development [70]; *FUL* (G3, first flower QTLs 4–1, 5–1, 12–2, 26–9, and 26–12), which functions early in controlling flowering time [71, 72]; *EMF1* (G3), which is involved in DTF through the regulation of reproductive development [73, 74]; *FKF1* (G3), involved in DTF by regulating changes in photoperiod and temperature [75, 76]; and *NF-YB8* (G3 and G5, pod maturity QTLs 14–2 and 15–2), which regulates leaf development and maturity [77]. The interaction groups G4 and G5 possessed the *E3* gene in common, together with one and two other genes, respectively (Fig 3a, Table 2), including *STM* (G4, first flower QTL 12–3), which is related to flower meristem and DTF [78]; *FLK* (G5, plant height QTL 26–17), which regulates DTF through the repression of *FLOWERING LOCUS C* expression and its post-transcriptional modification [76]; and *NF-YB8* (G5), also detected in G3, as described above.

The G6 and G7 interaction groups contained only eight non-significant genes identified in GWAS, but were detected as significant interaction groups on DTF in the epistatic analysis (Fig 3b, Table 2). These eight genes included *HAP5A* (G6, one plant height QTL and three pod maturity QTLs), related to earlier flowering [79]; *ARP6* (G6, eight first flower QTLs), involved in plant growth and development [80, 81]; *FUL* (G6), also identified in G3, as described above; *BBX15* (G6), indirectly related to light reaction [82]; *SWN* (G7), involved in the regulation of flowering and development [83, 84]; *FVE* (G7), associated with the regulation and control of DTF [85, 86]; and *PHYE* and *CRY2* (G7, two pod maturity QTLs and one plant height QTL), related to light reaction during flowering [87, 88].

### Pathways of epistatic genes related to DTF

**Fig 4:**
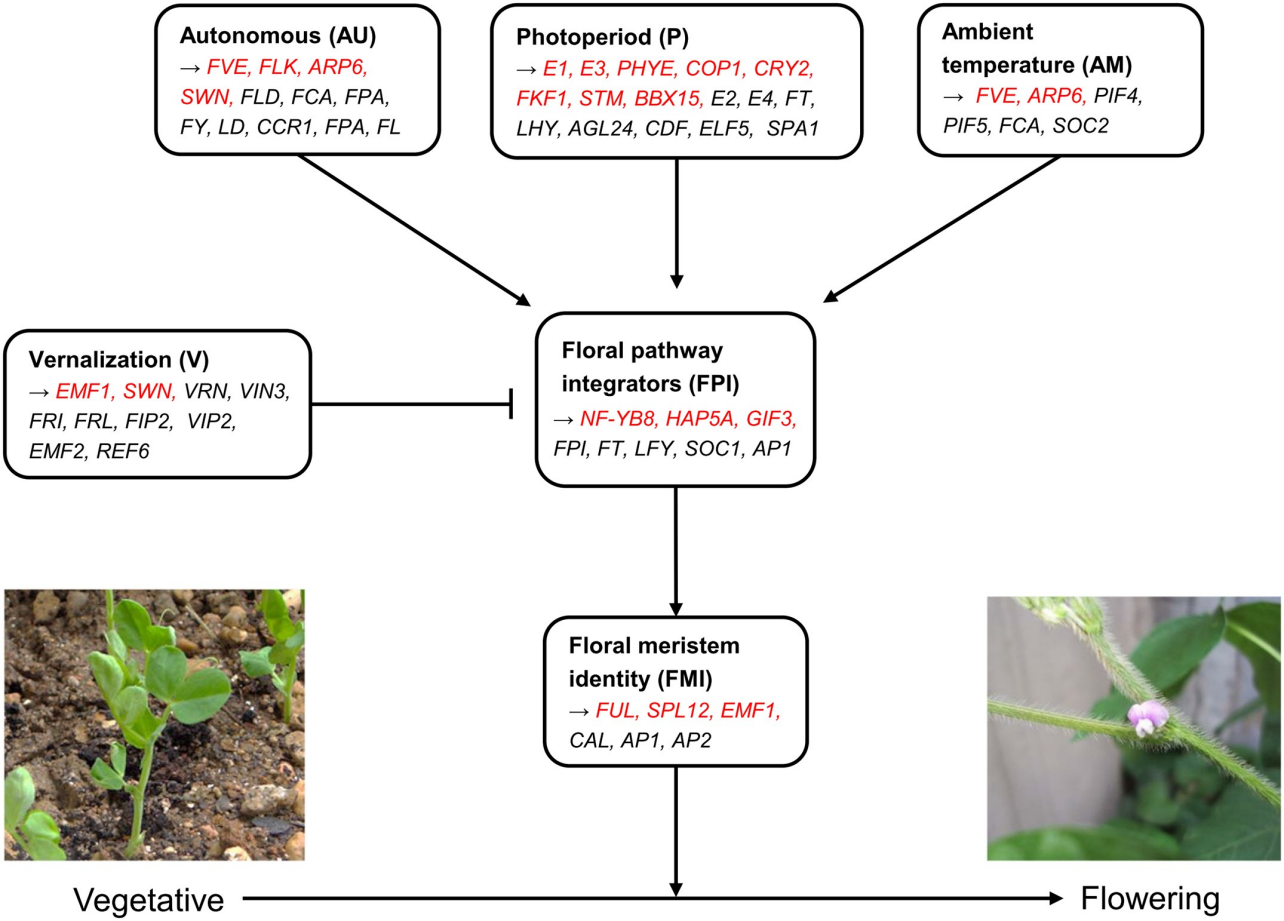
Genes in major pathways regulating plant growth from the vegetative stage to the flowering stage. Representative genes are displayed in corresponding pathways. Candidate genes identified from interaction groups are shown in red (“Related pathway” column in Table 2).

To identify the degree of involvement in DTF, two significant and 16 non-significant genes in all interaction groups were examined on DTF-related pathways. These 18 candidate genes were involved in the following six major DTF-related pathways [56]: ambient temperature (AM), autonomous (AU), flowering pathway integrator (FPI), photoperiod (P), floral meristem identity (FMI), and vernalization (V) (Table 2, Fig 4). The V and P pathways control the overall flowering process [89] and the AM pathway regulates flowering time [90, 91], as external factors. The AU pathway promotes induction of flowering as an internal factor [89]. The FPI pathway regulates the other pathways and triggers the induction of FMI [89], and the FMI pathway induces flowering. The P pathway contained eight candidate genes including the *E1* and *E3* genes; A and AM pathways contained two candidate genes each; AU and FPI pathways contained four candidate genes each; and the FMI pathway contained three candidate genes. These results confirmed that all 18 candidate genes are involved in major DTF-related pathways, and presented that they have a wide effect on DTF while interacting with each other.

## Discussion

The Axiom^®^ 180k SoyaSNP array was developed by our team mainly based on Korean soybean accessions, with the aim to perform GWAS using a large number of high-density markers [45]. Of the 2,662 soybean accessions used in this study (S1 Dataset), 94% were of Korean origin and therefore suitable for genotyping with the Axiom^®^ 180k SoyaSNP high-density array, generating 180,961 SNP markers. These 2,662 soybean accessions mainly comprised two subgroups, LRs and ICs, and have a tendency to form a single large population with considerable genomic diversity (Fig 1). The Fst value, a numeric value indicating population structure, was also sufficiently small (0.0581) for the two subgroups to be considered as a single group (S1 Table). Based on the results of population structure analyses, we conducted GWAS on all 2,662 soybean accessions and identified 59 soybean genes, including *E1* and E3, as candidates associated with DTF (Fig 2, S2 and S3 Datasets).

Genes *E1*–*E10* [31–41], *J* [42], and *Dt1* [43, 44] are known as major DTF-related genes in soybean. Among these, genes *E1*–*E4* act as the major determinants of DTF, as these genes account for approximately 62–66% of variation in DTF [40, 57]. The *E1* gene has the largest effect on DTF and photoperiod sensitivity [37, 63] through regulations of *GmFT2a*, *GmFT5a*, and *GmFT1a* genes [92, 93]. The *E2* gene enhances photoperiod response and contributes to early flowering by regulating *GmFT2a* [66, 94]. Genes *E3* and *E4* are involved in the regulation of *E1* and affect DTF through the regulation of the far-red light ratio [65, 67]. Above all, *E1* functions as a key regulator of DTF in soybean [95]. In our GWAS, *E1* and *E3* genes showed significant *p*-values, whereas *E2* and *E4* genes were not detected (Fig 2). The *E2* gene showed a non-significant *p*-value and the *E4* gene was excluded from the GWAS because of considerably low MAF of SNPs. According to previous studies, the *E1*–*E4* genes in soybean are considerably affected by habitat ecology and latitude [57, 96–98]. The soybean population used in this study consists mostly of Korean accessions and therefore reflects the Korean environment. Korean soybeans are also reported to possess high genomic diversity, unlike soybean accessions from other regions [99–101]. Considering the genomic characteristic of our population, the GWAS result suggests that *E1* and *E3* genes play a significant role in the regulation of DTF trait in Korean soybean population.

In addition to GWAS results, the interaction effects between 59 significant genes and the remaining non-significant genes on DTF were examined (Table 1). Identification of epistatic interactions could help in the detection of a greater number of candidate genes, since the complex DTF trait is affected by interactions among multiple genes [29, 102]. In our epistatic analysis, non-significant genes were utilized as 2,188 soybean genes which are homologous genes to 356 DTF-related *Arabidopsis* genes (S4 Dataset). This approach of marker reduction, called biological filtering, has been reported as one of the effective approaches for epistatic analysis [103, 104], as it allows the overcoming of computational limitations caused by a considerable number of marker combinations [105]. Therefore, we selected 356 *Arabidopsis* genes based on previous reports on DTF and maturity (S4 Dataset), and used to analyze the interaction effects of various DTF-related genes on DTF. The results of epistatic analysis revealed seven interaction groups and 18 candidate genes with significant influence on DTF (Table 2); *E1* and *E3* genes, which were detected in GWAS, also played a major role in the interaction groups (Fig 3).

DTF is regulated by complex networks of biological processes [106, 107], and is characterized to six representative pathways from vegetative to flowering stages in *Arabidopsis* [108, 109]: P (response to day length and light), V (cold exposure), AU (flowering promotion), FPI (floral transition), AM (time of floral transition), and FMI (floral development) (Fig 4). To better understand the genetic effects of candidate genes on DTF, it is necessary to identify the extent to which these candidate genes are involved in the six major pathways. Among the 18 candidate genes identified in this study, eight genes including *E1* (interaction groups G1–G3) are involved in P, FPI, FMI, and V pathways; four genes including *E3* (G4 and G5) are associated with P, AU, and FPI pathways; and eight genes (G6 and G7) are related to P, V, AU, AM, FMI, and FPI pathways (Table 2, Fig 4). Thus, all of these 18 candidate genes were evenly distributed in the six major pathways and affected DTF while interacting with each other in the corresponding interaction groups. Overall, our results of GWAS and epistatic analysis suggest that these 18 candidate genes play a significant role in the regulation of DTF in soybean, at least in the Korean population.

However, our findings have several limitations. First, the DTF-related candidate markers identified in this study do not represent the worldwide soybean population. It is possible that these candidate markers are specific to Korean soybean accessions because 94% of the soybean population was of Korean origin. Second, the epistatic analysis was restricted to only 356 DTF-related *Arabidopsis* genes. The 356 genes were used to reduce computational overload; however, markers excluded in the epistatic analysis could also have a significant effect on DTF while interacting with each other. Finally, our candidate markers have not been validated by biological experiments. To minimize this limitation, we examined the genomic characteristics of the soybean population before GWAS and used a conservative cut-off in GWAS, which was higher than the Bonferroni-adjusted *p*-value. Despite these efforts, our candidate markers need further experimental validation, but they are expected to present valuable information to soybean breeding programs aimed at improving the DTF trait.

## Conclusion

DTF is an important agronomic trait relevant to plant growth, development, and productivity in soybean. This trait is regulated by complex biological processes and is affected by endogenous genetic factors as well as environmental cues. Many researches have demonstrated the effect of genotype × environment interaction on DTF; however, studies on epistatic interactions among genetic factors have rarely been reported. Although our study is restricted by the origin of soybean accessions and also requires further biological validation, candidate markers identified in this study provides not only additional information for understanding the DTF trait in soybean but also a valuable genetic basis for soybean breeding programs aimed at improving DTF.

## Supporting information

S1 Dataset Sample information of origin, maturity group, and flowering time. (XLSX)

S2 Dataset List of soybean genes and number of SNP markers (MAF > 0.05) per gene. (XLSX)

S3 Dataset Results of GWAS on flowering time in 2,662 soybean accessions. (XLSX)

S4 Dataset List of DTF-related genes reported in *Arabidopsis thaliana*, and their homologous genes to soybean. (XLSX)

S1 Fig Ten-times cross-validation error values for population structure analysis from *K* = 2 to *K* = 8. (TIF)

S1 Table Average π, F, and LD values among soybean accessions. (DOCX)
