# Supplementary material for: Genome-wide association and epistatic interactions of flowering time in soybean cultivar
Source: PLoS One. 2020 Jan 22;15(1):e0228114. doi: 10.1371/journal.pone.0228114 (PMC6975553; doi:10.1371/journal.pone.0228114)
Supplement: S1 Table — (DOCX) [file pone.0228114.s006.docx]

Table S1. Average π, F, and LD values among soybean accessions

| Group | Sample  size | Average  π | Average  F | Average LD  r^2^ (100Kb) | Average LD  r^2^ (500Kb) |
| --- | --- | --- | --- | --- | --- |
| Cultivar | 2662 | 2.51E-05 | 0.97206 | 0.221143 | 0.119213 |
| IC | 335 | 2.24E-05 | 0.97106 | 0.232546 | 0.121583 |
| LR | 2175 | 2.40E-05 | 0.97371 | 0.227096 | 0.112253 |
